# Self-reported chewing or swallowing problems and episodic memory performance in older Americans: a longitudinal cohort study

**DOI:** 10.3389/fnagi.2026.1899130

**Published:** 2026-09-30

**Authors:** Fei He, Chonghao Yao, Xuteng Kang, Yihao Ding, Haoqi Tu, Sijia Na

**Affiliations:** 1 Key Laboratory of Shaanxi Province for Craniofacial Precision Medicine Research, College of Stomatology, Xi'an Jiaotong University, Xi'an, China; 2 Department of Oral and Maxillofacial Surgery, College of Stomatology, Xi'an Jiaotong University, Xi'an, China; 3 Robotics Engineering, School of Automation Science and Electrical Engineering, Beihang University, Beijing, China

**Keywords:** aging, chewing, dysphagia, episodic memory, longitudinal study, NHATS, swallowing

## Abstract

**Background:**

Impaired mastication is a plausible modifiable risk factor for dementia, but long-term longitudinal evidence using functional metrics instead of tooth counts is limited. This study investigated the association between self-reported chewing/swallowing problems and cognitive decline trajectories in older adults.

**Methods:**

We analyzed repeated NHATS rounds 7–14. The primary analytic sample comprised 8,636 participants contributing 34,571 person-round observations with valid exposure and immediate plus delayed 10-word recall. Raw total recall (0–20) was the primary outcome. Linear mixed models used rounds since each participant’s first complete analytic assessment, time-varying exposure, random intercepts and random slopes, and adjustment for age, sex, race/ethnicity, education, comorbidity count, depressive symptoms, hearing impairment, and vision impairment.

**Results:**

At the first complete analytic assessment, 842 participants (9.7%) reported chewing or swallowing problems. In the primary model, the exposure-by-time interaction was −0.035 recall points per follow-up round (95% CI, −0.075 to 0.005; *p* = 0.086). The contemporaneous exposure coefficient at follow-up round 0 was 0.034 points (95% CI, −0.106 to 0.173; *p* = 0.638). Results were similar when participants with one assessment were excluded and when the outcome was modeled as immediate recall, delayed recall, or standardized against a common reference.

**Conclusion:**

In this analytic NHATS sample, self-reported chewing or swallowing problems were not independently associated with a statistically significant difference in episodic-memory trajectory after sensory and other prespecified adjustment. The item may warrant oral, swallowing, functional, or neurological assessment, but it is not a specific marker of masticatory dysfunction or cognitive risk.

## Introduction

Cognitive change in later life is clinically important, but associations between everyday oral or eating difficulties and cognitive outcomes require careful interpretation. Functional questions can be useful for screening, yet their meaning depends on the exact wording and the clinical context in which they are reported (Livingston et al., 2024).

The NHATS exposure used here is a single self-reported question about problems with chewing or swallowing. Chewing impairment and swallowing impairment are clinically distinct. Chewing difficulties may be related to dentition, pain, oral motor function, or food texture; swallowing difficulty can reflect dysphagia, frailty, sarcopenia, stroke, medication effects, neurological disease, or neurodegenerative disease (Yang et al., 2022; Coelho-Junior et al., 2025). Because NHATS combines the two experiences, the present data cannot distinguish them or identify a specific oral mechanism.

Bidirectional relationships are also plausible. Cognitive or neurological impairment can contribute to eating and swallowing difficulties, while common causes such as age, education, multimorbidity, sensory impairment, frailty, and functional vulnerability can influence both the self-report and memory performance (Livingston et al., 2024; Weuve et al., 2015; Lin et al., 2011). Mechanistic hypotheses involving periodontal inflammation, tooth loss, cerebral perfusion, or trigeminal stimulation are not directly evaluated by this combined questionnaire and are therefore not treated as tested explanations in this study.

Longitudinal evidence using repeated functional self-reports remains limited. Dysphagia-focused work in older adults has linked swallowing difficulty to adverse health, frailty, nutrition-related concerns, and cognitive impairment, although longitudinal estimates have not consistently shown an independent association with subsequent health outcomes (Yang et al., 2022; Coelho-Junior et al., 2025). Recent studies in community and long-term-care settings likewise underscore that dysphagia may be a marker of broad vulnerability rather than a discrete dental exposure (Coelho-Junior et al., 2025; Formisano et al., 2025).

This study aimed to examine whether self-reported chewing or swallowing problems were associated with differences in repeated immediate and delayed word-recall performance among older adults participating in NHATS. We hypothesized that participants reporting problems would show lower observed episodic-memory performance and assessed, exploratorily, whether their adjusted memory trajectories differed over follow-up. We did not test mediation by nutritional status or make causal claims.

## Methods

### Design and data source

NHATS is a longitudinal study of Medicare beneficiaries aged 65 years or older in the United States (National Health and Aging Trends Study, 2026). We used delivered person-round records from rounds 7 through 14. The retained analytic extract did not include the survey design variables required for weighted variance estimation; all estimates in this report are therefore unweighted analytic-sample estimates, and we make no claim of national representativeness.

### Sample selection

We first identified 36,465 persons in the delivered round 7–14 file. We required a valid response to the chewing-or-swallowing question and valid immediate and delayed 10-word recall in at least one round. The primary analytic cohort contained 8,636 participants and 34,571 complete person-round observations. A participant could contribute one to eight assessments; 1,010 participants (11.7%) contributed a single complete assessment and thus informed levels but not within-person change. The reproducible selection sequence is shown in Supplementary Table S2 and assessment contribution in Supplementary Table S5; the corresponding analytic flow and interpretation framework are shown in Figure 1, and the contribution distribution is shown in Figure 2.

**Figure 1:**
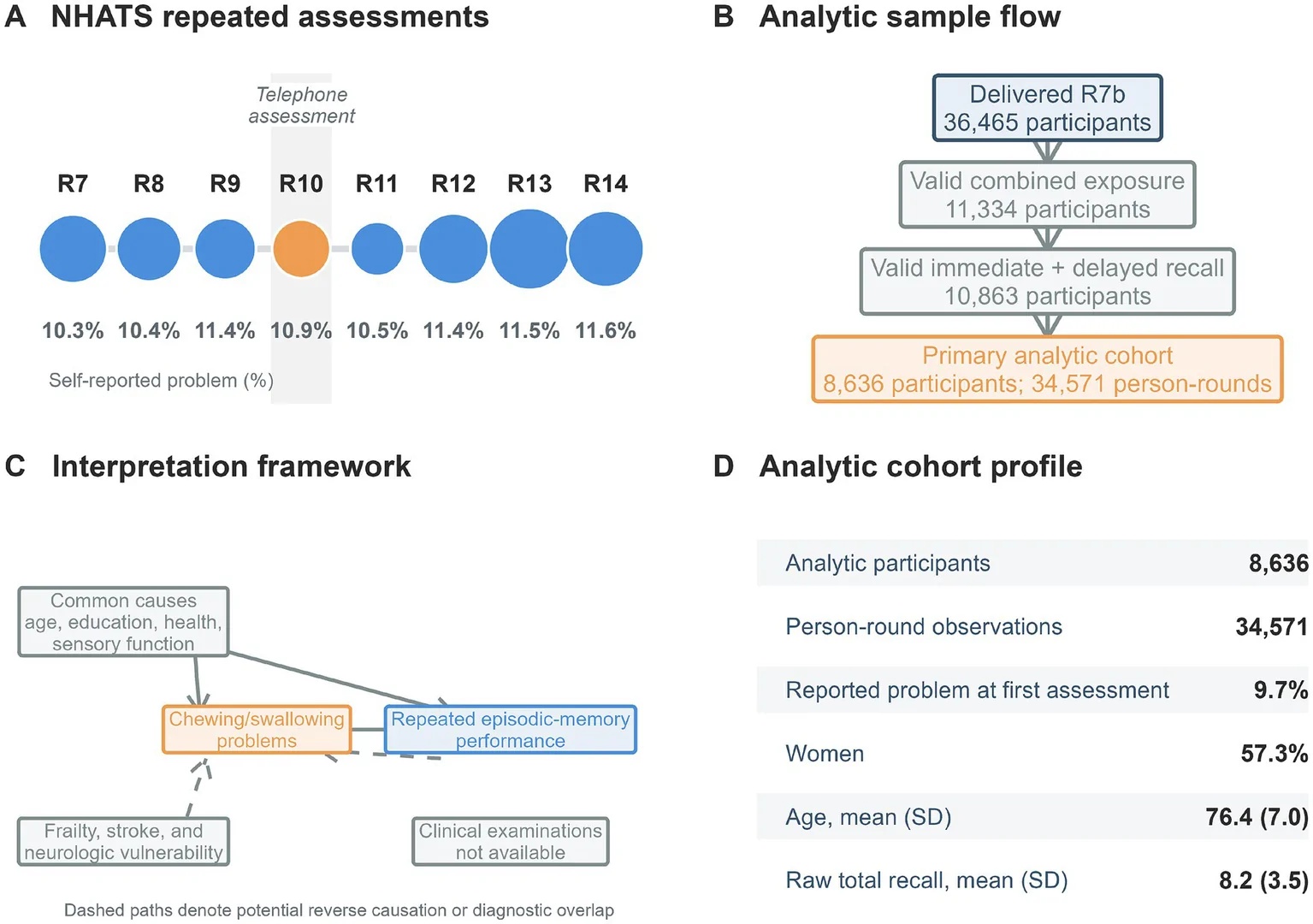
Study design, sample flow, and interpretation framework. **(A)** NHATS rounds 7–14; circle size reflects valid combined-exposure records and labels give unweighted reported-problem prevalence. **(B)** Selection from the delivered file to the primary analytic cohort. **(C)** The combined chewing-or-swallowing self-report can be influenced by common causes and reverse-causation pathways; clinical components cannot be separated in NHATS. **(D)** Basic unweighted analytic-sample profile at the first complete assessment.

**Figure 2:**
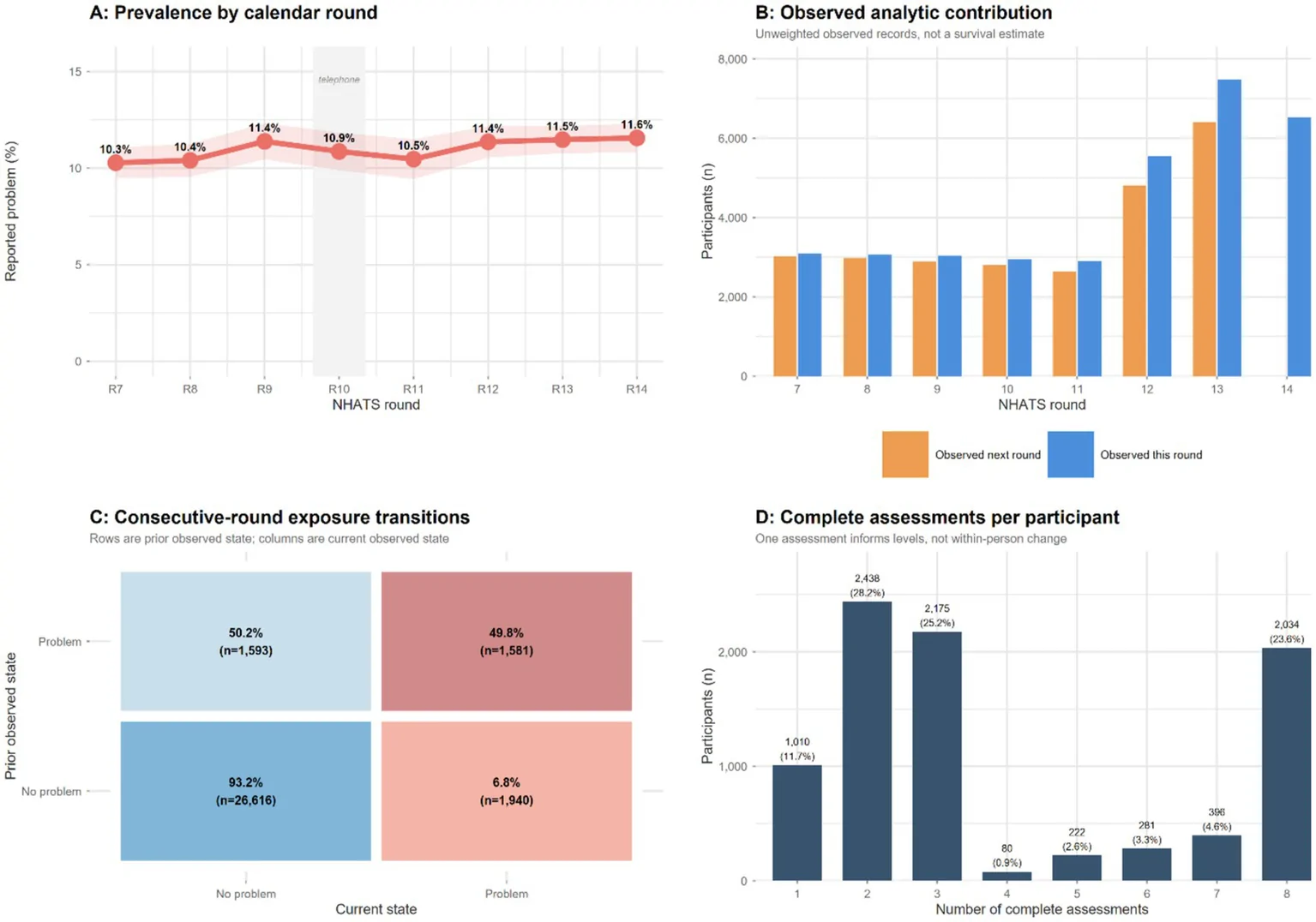
Epidemiology and observed longitudinal data structure. All panels are unweighted. **(A)** Reported-problem prevalence among valid exposure records by calendar round. **(B)** Complete analytic records observed at each round and also observed at the following round; this is not a survival estimate. **(C)** Consecutive observed exposure-state transitions. **(D)** Number of complete analytic assessments contributed per participant; participants with one assessment inform levels but not within-person change.

### Exposure

At each observed round, participants were classified as reporting or not reporting a problem on the NHATS question combining chewing and swallowing. The exposure was modeled as time varying. We consistently refer to this construct as self-reported chewing or swallowing problems; it does not separate mastication from dysphagia and should not be interpreted as a clinical diagnosis.

### Outcome

Immediate and delayed 10-word recall scores were each retained only when within their valid 0–10 range. Their raw sum (0–20) was the primary measure of episodic-memory performance. This use of raw recall preserves a common scale across rounds. Sensitivity analyses used immediate and delayed components separately, a score standardized to the round-7 raw-score distribution, and a within-round standardized score. The latter was explicitly treated as secondary because its reference distribution changes across rounds.

### Covariates and harmonization

Variables were harmonized across rounds using consistent coding rules (Supplementary Table S1). Age, sex, race/ethnicity, and education category were fixed at the first complete analytic assessment. Comorbidity count, PHQ-2 score, hearing impairment, and vision impairment were updated when observed. Hearing and vision were included as covariates rather than used as negative-control exposures, because they are plausible common causes and sensory impairment is associated with cognitive outcomes (Lin et al., 2011). Short Physical Performance Battery (SPPB) score and AD8 status were reserved for prespecified sensitivity analyses because of availability and conceptual concerns.

### Statistical analysis

The primary linear mixed model used follow-up round, defined as time since each participant’s first complete analytic assessment, rather than calendar round. It included current chewing-or-swallowing problem status, follow-up round, their interaction, the prespecified covariates, participant-specific random intercepts, and participant-specific random slopes for follow-up round with an estimated intercept-slope correlation. The current exposure coefficient is interpreted at follow-up round 0, not as a study-wide calendar baseline difference. The interaction estimates a difference in model-implied slope while participants may change exposure status and is not converted to an equivalent number of years of cognitive aging.

### Robustness analyses

The interaction estimates the difference in model-implied slopes while allowing exposure status to vary over time; it was not converted to an equivalent number of years of cognitive aging. Complementary models used baseline exposure, one-round-lagged exposure, and within-person/between-person exposure decomposition. These analyses address different estimands and are not treated as confirmatory evidence for causality.

## Results

### Analytic cohort

Of 8,636 participants, 842 (9.7%) reported a chewing or swallowing problem at their first complete analytic assessment. Compared with participants without a reported problem, this group was older, had lower education, more comorbidities and depressive symptoms, more hearing and vision impairment, lower SPPB scores, and lower observed immediate, delayed, and total recall scores (Table 1). Unweighted prevalence, observed follow-up contribution, consecutive-round exposure transitions, and complete-assessment contribution are shown in Figure 2. Unadjusted immediate, delayed, and total raw-recall patterns are shown in Figure 3. These descriptive differences do not establish an independent longitudinal association.

**Table 1:** Characteristics at the first complete analytic assessment, by self-reported chewing or swallowing problem status

Characteristic	No problem	Problem	*p* value
Participants, *n*	7,794	842	
Age, mean (SD), years	76.25 (6.92)	77.40 (7.46)	<0.001
Female, *n* (%)	4,430 (56.8%)	515 (61.2%)	0.018
Race/ethnicity, *n* (%)			<0.001
Non-Hispanic White	4,730 (60.7%)	436 (51.8%)	
Non-Hispanic Black	1,512 (19.4%)	147 (17.5%)	
Hispanic	1,270 (16.3%)	217 (25.8%)	
Other	282 (3.6%)	42 (5.0%)	
Education category, median (IQR)	6 (4, 8)	4 (3, 6)	<0.001
Comorbidity count, mean (SD)	1.56 (1.61)	2.46 (2.08)	<0.001
PHQ-2 score, mean (SD)	2.84 (1.29)	3.71 (1.70)	<0.001
Hearing impairment, *n* (%)	795 (10.2%)	211 (25.1%)	<0.001
Vision impairment, *n* (%)	397 (5.1%)	107 (12.7%)	<0.001
SPPB score, mean (SD)	6.97 (3.12)	5.17 (3.21)	<0.001
AD8-positive, *n* (%)	10 (0.1%)	1 (0.1%)	1
Immediate recall, mean (SD)	4.88 (1.67)	4.39 (1.69)	<0.001
Delayed recall, mean (SD)	3.43 (2.05)	3.00 (2.01)	<0.001
Total recall (0–20), mean (SD)	8.31 (3.45)	7.38 (3.41)	<0.001

All values are unweighted. Values are *n* (%), mean (SD), or median (IQR). *p* values compare the two groups descriptively and are not causal estimates.

AD8, 8-item informant interview to differentiate aging and dementia; PHQ-2, 2-item Patient Health Questionnaire; SPPB, Short Physical Performance Battery. The exposure combines chewing and swallowing problems and does not diagnose mastication impairment or dysphagia.

**Figure 3:**
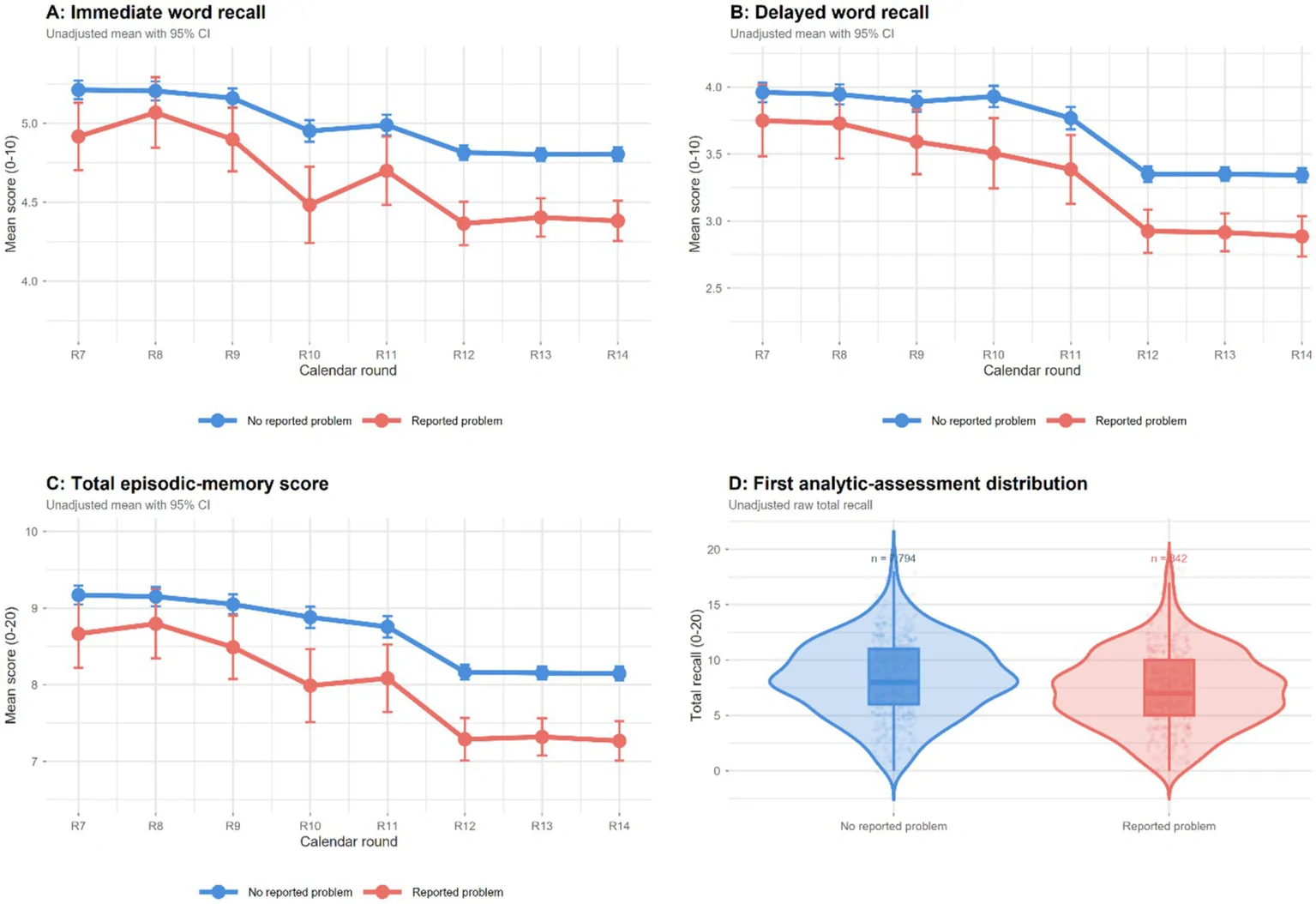
Observed immediate, delayed, and total recall performance. All panels are unadjusted. **(A–C)** Mean raw immediate, delayed, and total recall by calendar round and current reported-problem status; bars are 95% confidence intervals. **(D)** Raw total-recall distribution at the first complete analytic assessment. These descriptive patterns are not adjusted longitudinal estimates.

### Primary longitudinal model

In the prespecified sensory-adjusted mixed model, total recall declined by −0.106 points per follow-up round in the reference exposure group (95% CI, −0.122 to −0.089; *p* < 0.001). At follow-up round 0, current reported problems were not associated with a difference in total recall (beta = 0.034, 95% CI, −0.106 to 0.173; *p* = 0.638). The problem-by-follow-up-round interaction was −0.035 points per round (95% CI, −0.075 to 0.005; *p* = 0.086), which did not meet the conventional two-sided 0.05 threshold (Table 2; Supplementary Table S3; Figure 4). Random-intercept and random-slope standard deviations were 2.111 and 0.199 points, respectively, with an intercept-slope correlation of 0.165.

**Table 2:** Primary sensory-adjusted mixed model for raw episodic-memory performance

Term	Estimate (95% CI)	*p* value
Current reported chewing or swallowing problem at follow-up round 0	0.034 (−0.106 to 0.173)	0.638
Follow-up round	−0.106 (−0.122 to −0.089)	<0.001
Reported problem × follow-up round	−0.035 (−0.075 to 0.005)	0.086

Outcome: immediate plus delayed 10-word recall, 0–20. The table reports the three prespecified exposure/time terms from the primary model; the full covariate model and random-effects parameters are in Supplementary Table S3.

Model specification. *n* = 8,636 participants and 34,571 complete person-round observations. Time is rounds since each participant's first complete analytic assessment. Current chewing-or-swallowing problem status is time varying. The model includes age, sex, race/ethnicity, education category, comorbidity count, PHQ-2 score, hearing impairment, vision impairment, participant-specific random intercepts, and participant-specific random slopes. The current-exposure coefficient is evaluated at follow-up round 0; it is not a calendar-baseline contrast.

**Figure 4:**
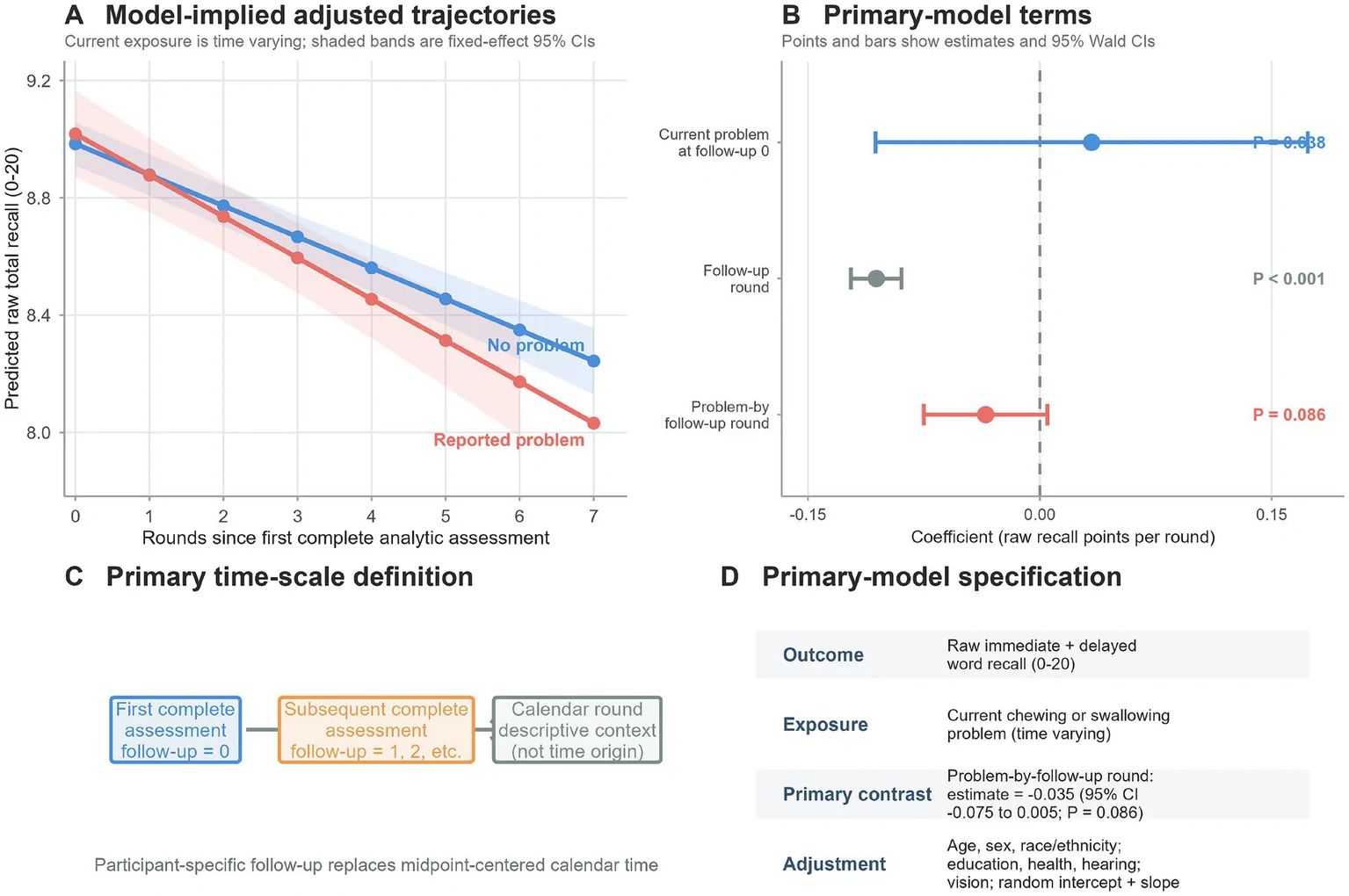
Primary sensory-adjusted longitudinal model. **(A)** Model-implied raw total-recall trajectories from the primary mixed model. Current exposure is time varying; ribbons are fixed-effect 95% confidence intervals. **(B)** Primary-model terms. **(C)** Participant-specific follow-up time used in the primary model. **(D)** Prespecified outcome, exposure, contrast, and adjustment set. The primary interaction estimate is −0.035 recall points per follow-up round (95% CI, −0.075 to 0.005; *p* = 0.086).

### Sensitivity and complementary analyses

The interaction estimates were similar and nonsignificant among participants with at least two assessments (−0.034; 95% CI, −0.075 to 0.007), after excluding round 10 (−0.039; 95% CI, −0.079 to 0.001), and after additional SPPB adjustment (−0.028; 95% CI, −0.071 to 0.015). Immediate- and delayed-recall models were likewise nonsignificant. Common-reference standardization yielded −0.010 standard deviations per round (95% CI, −0.021 to 0.001). The within-round standardized sensitivity estimate was −0.008 (95% CI, −0.019 to 0.003; Supplementary Table S4; Figure 5).

**Figure 5:**
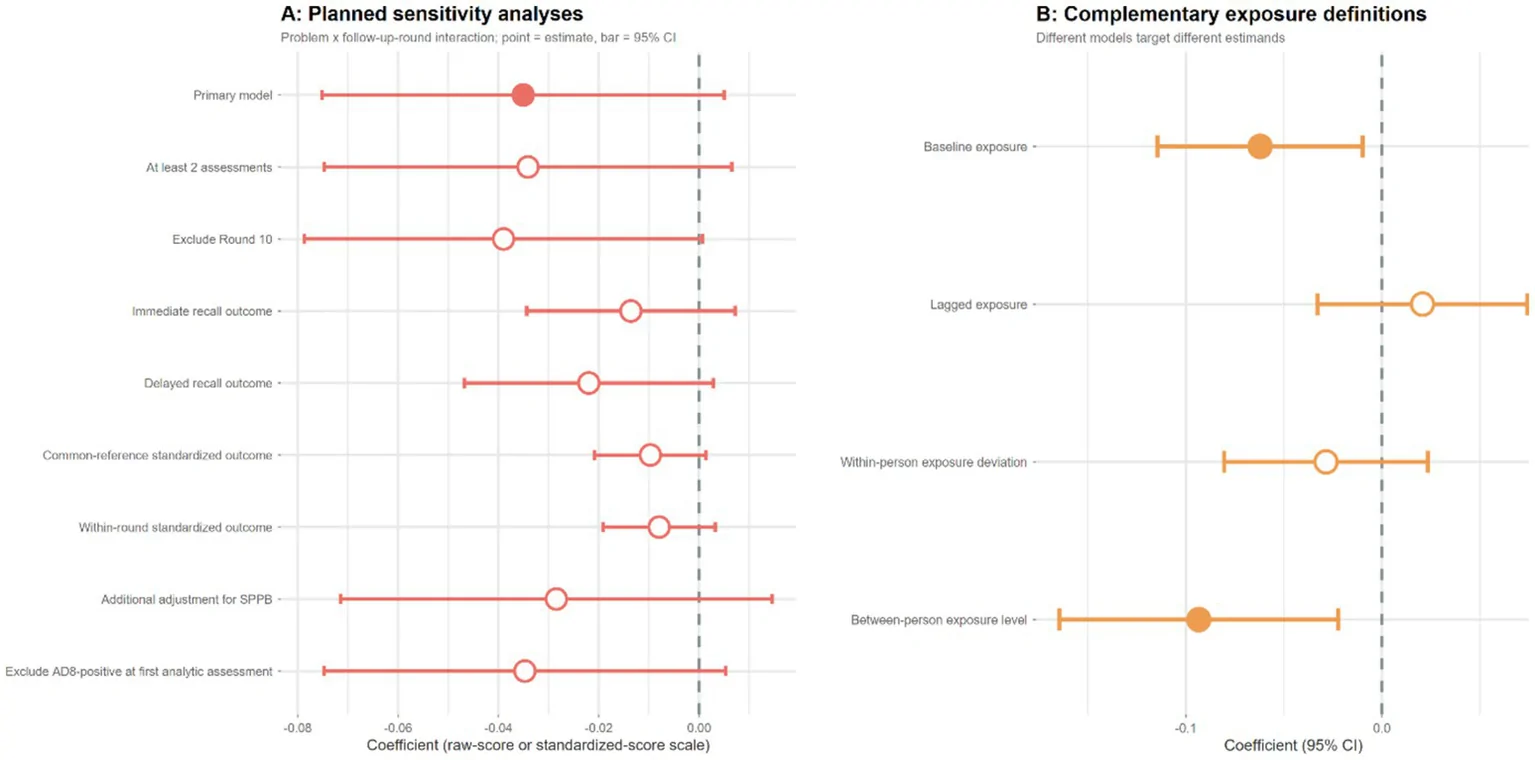
Robustness checks and complementary exposure definitions. **(A)** Planned outcome, sample, and adjustment sensitivities. **(B)** Baseline, lagged, within-person, and between-person exposure definitions. Points are estimates and bars are 95% Wald confidence intervals. The complementary models target different estimands and are not causal-effect estimates.

The baseline-exposure model yielded a nominal negative interaction (−0.062 points per round; 95% CI, −0.114 to −0.010), whereas the lagged-exposure and within-person exposure-deviation models were not significant. The between-person component was negative (−0.093; 95% CI, −0.164 to −0.022). Because these models address distinct questions and remain susceptible to residual confounding and selection, they are presented as complementary descriptive analyses rather than evidence of a causal effect (Supplementary Table S4; Figure 5).

## Discussion

In this repeated-observation analysis, self-reported chewing or swallowing problems were associated with lower unadjusted episodic-memory performance at the first complete analytic assessment, but the primary adjusted exposure-by-time interaction was not statistically significant. The primary finding is therefore not evidence that the combined self-report independently predicts accelerated episodic-memory decline.

The combined wording is central to interpretation. The item may capture chewing difficulties, dysphagia, or both, and swallowing difficulty may occur with frailty, stroke, sarcopenia, neurological disease, or early neurodegenerative change. These clinical pathways differ from dental or mastication-specific explanations. The data contain neither clinical oral examinations nor swallowing assessments, so periodontal disease, tooth loss, prosthodontic rehabilitation, cerebral perfusion, and oral inflammation cannot be invoked as directly measured mechanisms.

The attenuation of the association after inclusion of hearing impairment is informative but should not be overinterpreted. Hearing impairment is a plausible risk factor and common cause of poorer cognitive-test performance and health vulnerability, not a suitable negative-control exposure (Lin et al., 2011). Its inclusion, together with vision and other covariates, means the observed data do not support a claim of a robust association independent of sensory function.

The complementary baseline and between-person estimates may reflect persistent differences in health, social conditions, access to care, functional vulnerability, or pre-existing neurological disease. The null lagged and within-person estimates provide no simple resolution of reverse causation. Selective attrition, mortality, and cohort replenishment could also affect longitudinal comparisons. Our raw-score primary outcome avoids the changing reference distribution inherent in separate within-round standardization, but missing data and loss to follow-up remain important limitations.

This study has several strengths, including repeated observations, explicit separation of calendar round from participant-specific follow-up time, a raw episodic-memory outcome, and an integrated sample-selection and robustness record. Limitations include the single combined self-report, absence of clinical oral/swallowing assessments, potential exposure misclassification, residual confounding, incomplete design information for weighting, selective retention, and plausible bidirectionality. Food insecurity was not used as a proxy for nutritional status, and no mediation analysis was performed.

Clinically, a positive combined question may justify further oral, swallowing, functional, or neurological assessment according to the individual’s presentation. It should not be used alone as a specific marker of masticatory dysfunction or of cognitive risk. Future work should separately assess mastication and dysphagia, obtain clinically validated measures, define the temporal ordering of symptoms and cognition, and address attrition with appropriate survey-design information.

## Conclusion

Among older adults in this unweighted analytic NHATS sample, self-reported chewing or swallowing problems were not independently associated with a statistically significant difference in repeated raw word-recall trajectory after prespecified sensory and health adjustment. The combined screening question should be interpreted as a nonspecific indicator that may prompt further evaluation rather than as evidence of masticatory dysfunction or accelerated cognitive aging.

## Data Availability

Publicly available datasets were analyzed in this study. NHATS data are available to registered users from the National Health and Aging Trends Study at https://www.nhats.org/ under its data-use terms. Analysis code and derived tabular source data are provided in the Supplementary material.
