# Supplementary material for: Self-reported chewing or swallowing problems and episodic memory performance in older Americans: a longitudinal cohort study
Source: Front Aging Neurosci. 2026 Sep 30;18:1899130. doi: 10.3389/fnagi.2026.1899130 (PMC13661535; doi:10.3389/fnagi.2026.1899130)
Supplement: Supplementary file 1 [file Data_sheet_1.docx]

Supplementary eTables

Self-Reported Chewing or Swallowing Problems and Episodic-Memory Performance in Older Americans: A Longitudinal Cohort Study

eTable S1. Variable Harmonization and Analytic Coding

| **Construct** | **Retained source field(s)** | **Coding in revision** | **Analytic role** | **Time handling** | **Missing-value handling** |
| --- | --- | --- | --- | --- | --- |
| Self-reported chewing or swallowing problems | chew_swallow | 1=reported problem; 2=no reported problem; all other codes=missing | Primary exposure | Time varying | Incomplete records excluded from the primary complete-case record set |
| Immediate word recall | imm_recall | Valid integers 0-10 | Primary-outcome component | Time varying | Negative and out-of-range codes set to missing |
| Delayed word recall | dely_recall | Valid integers 0-10 | Primary-outcome component | Time varying | Negative and out-of-range codes set to missing |
| Raw total recall | imm_recall plus dely_recall | Sum of valid immediate and delayed scores (0-20) | Primary episodic-memory outcome | Time varying | Missing if either component missing |
| Age | age | Numeric age in years | Primary covariate | Fixed at first complete analytic assessment | Missing records excluded from primary model |
| Female sex | gender | 2=female; 1=male | Primary covariate | First valid report carried within participant | Missing records excluded from primary model |
| Race/ethnicity | race_hisp | 1=Non-Hispanic White; 2=Non-Hispanic Black; 4=Hispanic; 3/5/6=Other | Primary covariate | First valid report carried within participant | Missing records excluded from primary model |
| Education category | education | Valid codes 1-9 | Primary covariate | Fixed at first complete analytic assessment | Missing records excluded from primary model |
| Comorbidity count | comorb | Valid range 0-10 | Primary covariate | Time varying | Missing records excluded from primary model |
| Depressive symptoms | phq2 | Valid range 2-8 | Primary covariate | Time varying | Missing records excluded from primary model |
| Hearing impairment | hear_radiow | 2=impaired; 1=not impaired | Primary covariate | Time varying | Missing records excluded from primary model |
| Vision impairment | vision_close | 2=impaired; 1=not impaired | Primary covariate | Time varying | Missing records excluded from primary model |
| Physical performance | sppb | Valid range 0-12 | Sensitivity covariate | Time varying | Used only in the SPPB sensitivity model |
| Probable cognitive impairment | ad8_dem | 1=AD8-positive; -1=not positive | Sensitivity restriction | Status at first analytic assessment | Used only in the AD8-exclusion sensitivity model |
| Calendar round | round | Round 7-14 | Descriptive/attrition context | Time varying | Retained when observed |
| Individual follow-up time | round | Round minus each participant's first complete analytic round | Primary time scale | Time varying | Defined only for primary complete analytic records |

The combined NHATS question does not distinguish chewing impairment from swallowing impairment. No causal mediation, E-value, negative-control, or unvalidated oral-impairment-score analysis was retained.

eTable S2. Analytic Sample Selection

| **step** | **n_participants** | **n_person_rounds** |
| --- | --- | --- |
| Unique persons in delivered R7-R14 analytic file | 36465 | 195689 |
| At least one valid chewing/swallowing response | 11334 | 43134 |
| At least one valid immediate and delayed recall pair | 10863 | 40833 |
| At least one complete primary-model record | 8636 | 34571 |
| Primary analytic person-round records |  | 34571 |

The final cohort contains 8,636 unique participants and 34,571 complete person-round observations. Person-round records and persons are intentionally distinguished.

eTable S3. Fixed Effects From the Primary Mixed Model

| **term** | **Estimate** | **Std. Error** | **df** | **t value** | **Pr(>\|t\|)** |
| --- | --- | --- | --- | --- | --- |
| (Intercept) | 16.087 | 0.307 | 12211 | 52.433 | <0.001 |
| chew_problem | 0.034 | 0.071 | 26201 | 0.47 | 0.638 |
| followup_round | -0.106 | 0.008 | 4987 | -12.692 | <0.001 |
| age | -0.125 | 0.004 | 12498 | -33.897 | <0.001 |
| female | 1.022 | 0.055 | 8243 | 18.485 | <0.001 |
| race_ethnicityNon-Hispanic Black | -1.419 | 0.072 | 8093 | -19.598 | <0.001 |
| race_ethnicityHispanic | -1.452 | 0.081 | 8940 | -17.844 | <0.001 |
| race_ethnicityOther | -1.279 | 0.147 | 8736 | -8.693 | <0.001 |
| education_category | 0.439 | 0.013 | 8388 | 34.188 | <0.001 |
| comorbidity_count | -0.178 | 0.013 | 30261 | -14.202 | <0.001 |
| phq2_score | -0.118 | 0.013 | 34311 | -9.348 | <0.001 |
| hearing_impairment | -0.236 | 0.05 | 34014 | -4.75 | <0.001 |
| vision_impairment | -0.312 | 0.068 | 34213 | -4.612 | <0.001 |
| chew_problem:followup_round | -0.035 | 0.02 | 17543 | -1.716 | 0.086 |

Primary outcome: raw total immediate plus delayed recall (0-20). Time is rounds since each participant's first complete analytic assessment. Model includes participant-specific random intercepts and slopes.

eTable S3 (continued). Random-Effects Parameters

| **grp** | **var1** | **var2** | **vcov** | **sdcor** |
| --- | --- | --- | --- | --- |
| spid | (Intercept) |  | 4.457 | 2.111 |
| spid | followup_round |  | 0.039 | 0.199 |
| spid | (Intercept) | followup_round | 0.069 | 0.165 |
| Residual |  |  | 4.405 | 2.099 |

Random-effects specification is reported to reconcile the primary model description and results.

eTable S4. Prespecified Sensitivity Analyses

| **Analysis** | **Interaction estimate (95% CI)** | **P value** | **Participants** | **Person-round observations** | **Interpretation** |
| --- | --- | --- | --- | --- | --- |
| Primary model | -0.035 (-0.075 to 0.005) | 0.086 | 8636 | 34571 | Primary sensory-adjusted model. |
| At least 2 assessments | -0.034 (-0.075 to 0.007) | 0.1 | 7626 | 33561 | Participants with one assessment cannot inform within-person change. |
| Exclude Round 10 | -0.039 (-0.079 to 0.001) | 0.054 | 8635 | 31630 | Excludes the telephone-interview round. |
| Immediate recall outcome | -0.014 (-0.034 to 0.007) | 0.2 | 8636 | 34571 | Raw immediate 10-word recall score. |
| Delayed recall outcome | -0.022 (-0.047 to 0.003) | 0.082 | 8636 | 34571 | Raw delayed 10-word recall score. |
| Common-reference standardized outcome | -0.010 (-0.021 to 0.001) | 0.086 | 8636 | 34571 | Standardized using the Round-7 raw total-recall distribution. |
| Within-round standardized outcome | -0.008 (-0.019 to 0.003) | 0.163 | 8636 | 34571 | Sensitivity only; the outcome reference changes across rounds. |
| Additional adjustment for SPPB | -0.028 (-0.071 to 0.015) | 0.195 | 8170 | 27865 | Functional-status sensitivity; SPPB was unavailable for some primary records. |
| Exclude AD8-positive at first analytic assessment | -0.035 (-0.075 to 0.005) | 0.089 | 8625 | 34558 | Addresses possible prevalent cognitive impairment. |

All estimates are exposure-by-follow-up-round interactions unless otherwise labelled. Within-round standardization is sensitivity-only because its reference distribution changes by calendar round. The full machine-readable model-output fields are retained in eTable_S4_Sensitivity_Analyses.csv.

eTable S4 (continued). Complementary Exposure Definitions

| **Analysis** | **Interaction estimate (95% CI)** | **P value** | **Participants** | **Person-round observations** | **Interpretation** |
| --- | --- | --- | --- | --- | --- |
| Baseline exposure | -0.062 (-0.114 to -0.010) | 0.02 | 8636 | 34571 | Status fixed at first complete analytic assessment. |
| Lagged exposure | 0.021 (-0.033 to 0.074) | 0.448 | 7594 | 25581 | Exposure reported at the preceding observed annual round. |
| Within-person exposure deviation | -0.028 (-0.080 to 0.023) | 0.283 | 8636 | 34571 | Deviation from each participant's mean exposure status. |
| Between-person exposure level | -0.093 (-0.164 to -0.022) | 0.01 | 8636 | 34571 | Participant mean exposure level across observed assessments. |

Baseline, lagged, within-person, and between-person parameterizations target different estimands and should not be interpreted as mutually confirming causal analyses. The full machine-readable model-output fields are retained in eTable_S4_Alternative_Exposure_Analyses.csv.

eTable S5. Number of Complete Analytic Assessments Per Participant

| **n_assessments** | **n_participants** | **percent** |
| --- | --- | --- |
| 1 | 1010 | 11.7 |
| 2 | 2438 | 28.2 |
| 3 | 2175 | 25.2 |
| 4 | 80 | 0.9 |
| 5 | 222 | 2.6 |
| 6 | 281 | 3.3 |
| 7 | 396 | 4.6 |
| 8 | 2034 | 23.6 |

Participants with one assessment contribute to estimated levels but cannot contribute within-person outcome change.

The revision package also includes source data for each figure and analysis table.
